# Supplementary material for: Current Switching of Topological Spin Chirality in the van der Waals Antiferromagnet Co1/3TaS2
Source: Adv Mater. 2026 Mar 6;38(19):e22943. doi: 10.1002/adma.202522943 (PMC13040512; doi:10.1002/adma.202522943)
Supplement: Supplementary file 1 — Supporting File: adma72759‐sup‐0001‐SuppMat.docx. [file ADMA-38-e22943-s001.docx]

Supporting Information

**Current Switching of Topological Spin Chirality in the van der Waals Antiferromagnet Co_1/3_TaS_2_**

*Kai-Xuan Zhang,** *Seungbok Lee, Woonghee Cho, and Je-Geun Park**

K.-X.Z. and S.L. contributed equally to this work.

**Supporting Note 1. The novelty, challenges, distinctions, and significance of the present work.**

For clarity, we discuss the novelty, challenges, distinctions, and significance of our work logically from our starting premise to the final discoveries:

1. **Novel focus on “switching spin chirality in exotic topological 3Q antiferromagnet”:**

Our primary object is to control and switch a *topological order parameter*—the scalar spin chirality (or equivalently, the emergent real-space gauge flux)—in an *exotic topological 3Q antiferromagnet*. This focus is novel and distinct from most SOT studies in the literature.

1. **The challenge of electrically switching an antiferromagnet:**

The van der Waals Co_1/3_TaS_2_ antiferromagnet, which has attracted significant interest, hosts a topological spin chirality with unique magnetic topology. This multi-spin quantity can be measured via the topological Hall effect. Therefore, we propose the concept of current switching topological spin chirality based on Co_1/3_TaS_2_.

Our experiment requires switching the spins, since the spin texture governs the spin chirality. Therefore, the concept target can be broken down into two parts: how to control and switch the complex spin arrangements of an exotic antiferromagnet. Antiferromagnetic spintronics offers several advantages: zero stray field, faster dynamics, and stability against magnetic perturbations, which enable higher density, scalability, speed, and stability for next-generation memory devices and in-memory computing. However, due to the strong internal field and negligible coupling to external perturbations, switching an antiferromagnet remains a significant challenge despite considerable efforts in the field.

1. **The challenge of predicting spin chirality switching in complex antiferromagnet Co_1/3_TaS_2_:**

To address the above challenge, we proposed current-driven spin-orbit torque (SOT) mechanism to switch the complex antiferromagnet Co_1/3_TaS_2_. It is worth noting that while SOT has often been used to switch magnetic moments coherently in uniform ferromagnets, extending this to an antiferromagnet with a complex multi-spin structure is not straightforward. For example, in the topological 3Q state of the exotic antiferromagnet Co_1/3_TaS_2_, local moments point along multiple noncoplanar directions; therefore, a current-induced spin accumulation or spin polarisation cannot trivially generate a uniform effective torque. Since existing SOT models are largely based on uniform ferromagnets (e.g., the Fe_3_GeTe_2_ family), it was not *a priori* obvious that current-driven SOT would successfully switch the magnetic moments of a complicated antiferromagnet. This open question motivated our experiment: Can an applied current achieve reproducible, non-volatile, and efficient magnetic moment switching, thereby producing equivalent spin-chirality switching? Before our real experimental demonstration, such a possibility had not been established for Co_1/3_TaS_2_.

1. **Novel and efficient “intrinsic-self-SOT + field-free switching” for antiferromagnet:**

In sharp contrast to conventional SOT induced by heavy metals such as Pt, our group previously demonstrated the intrinsic self-SOT in the vdW uniform ferromagnet Fe_3_GeTe_2_. However, that work and subsequent studies by others reported only the intrinsic self-SOT in ferromagnets, and the switching of magnetic moments by self-SOT typically requires an assisting in-plane or out-of-plane magnetic field.

Our present work on Co_1/3_TaS_2_ demonstrates, for the first time, the self-SOT in an exotic complex antiferromagnet. More importantly, the self-SOT switching (together with topological spin chirality switching) occurs without an external magnetic field in a single antiferromagnet. The self-SOT switching is non-volatile, reproducible, reversible, and highly energy-efficient: the switching current density (1.8×10^6^ A/cm²) is competitive with, or even lower than, the most efficient SOT systems reported to date. This highlights our contributions to the fields of SOT and antiferromagnetic spintronics, beyond the specific realisation of chirality switching.

1. **Realisation of “current switching topological spin chirality”:**

Spin chirality and magnetic topology are fundamental concepts connecting these highly interesting topics: noncoplanar spin textures, real-space Berry phases, and the topological Hall effect. A longstanding challenge for both fundamental understanding and device application has been the electrical switching of topological spin chirality and its emergent gauge flux.

In this work, we introduce the concept of current-switching spin chirality and experimentally realise it using the vdW antiferromagnet Co_1/3_TaS_2_. We demonstrate that intrinsic SOT can electrically switch the topological spin chirality purely by current, without the need for heavy metals or a magnetic field. Our results exhibit clear, nonvolatile, reversible, and highly energy-efficient current-induced chirality switching, facilitated by its non-centrosymmetric lattice, Berry-curvature-rich electronic structure, and topological magnetism. This work establishes a novel framework for electrically generating, controlling, and exploiting topological spin chirality, which may apply to other skyrmion-hosting and chirality-driven systems.

In summary, our work provides a novel demonstration of the electrical writing of the chiral 3Q antiferromagnetic state in Co_1/3_TaS_2_. We believe it lays an important new foundation for future work combining vdW magnets, antiferromagnetic spintronics, SOT, and topological spin chirality.

**Supporting Figures**


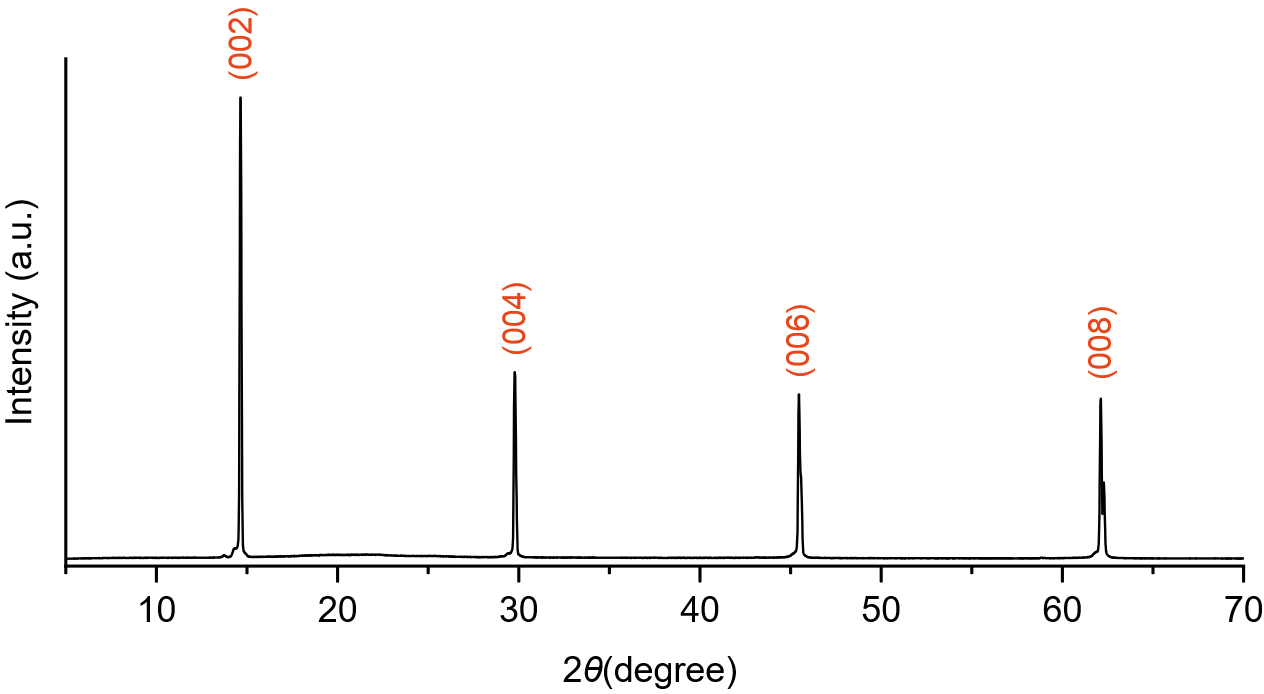


**Figure S1.** X-ray diffraction (XRD) pattern of our Co_1/3_TaS_2_ single crystal. XRD measurement was performed at room temperature using Rigaku Miniflex II, with the crystal aligned along the (001) plane. The sharp peaks of (002n) with n=1, 2, 3, 4 confirm the high crystalline quality of our crystal.


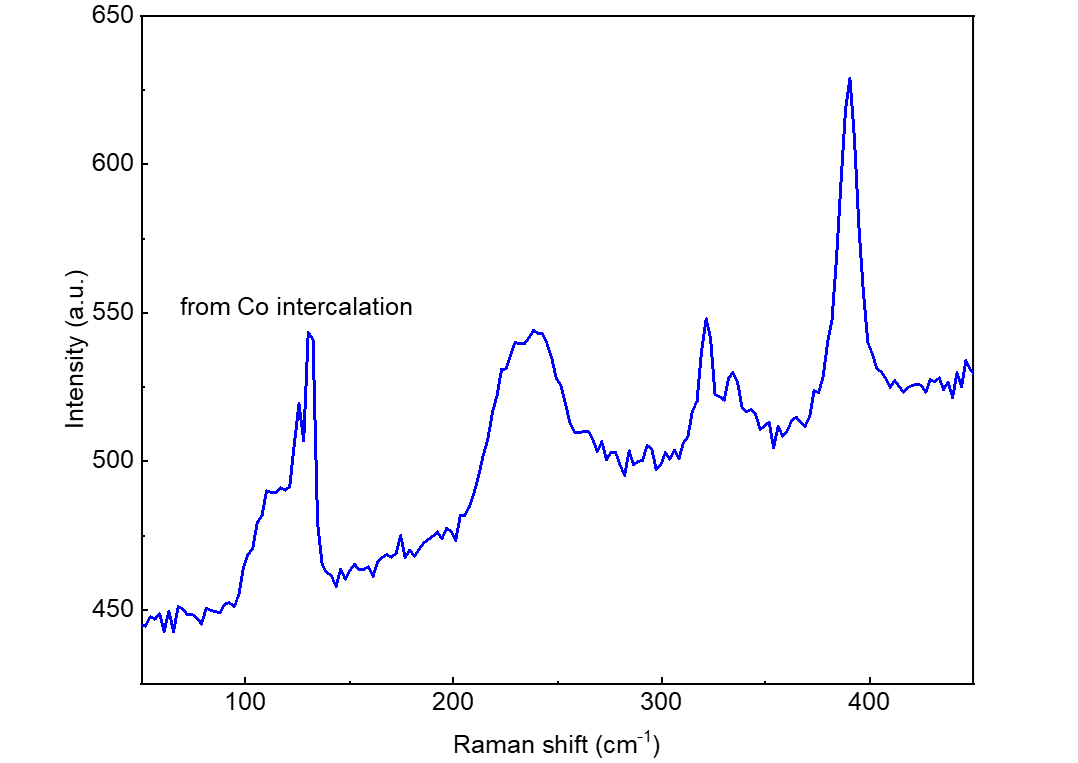


**Figure S2.** Raman spectra of a Co_1/3_TaS_2_ single crystal at room temperature. The Raman peak at around 137 cm^-1^ originates from Co intercalation, as indicated, demonstrating well-arranged Co intercalation and the high quality of the crystals.


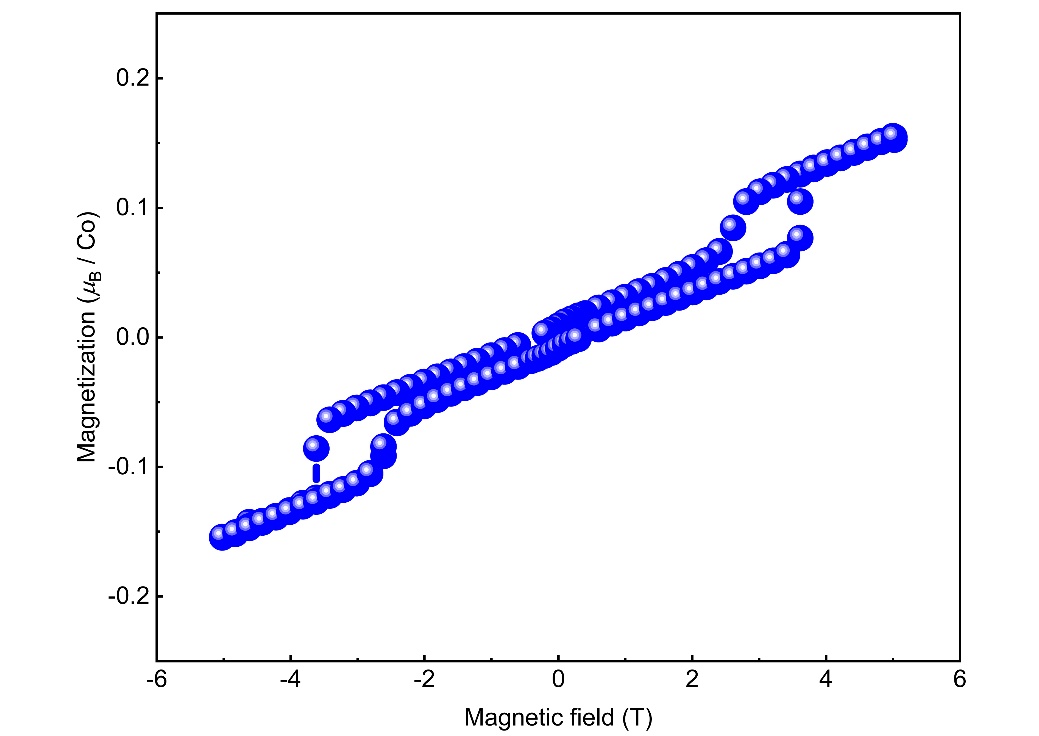


**Figure S3.** Magnetisation versus out-of-plane magnetic field (*M*-*H_z_* curve). The magnetic moment is small (0.1 μB/Co) at a high magnetic field and, most importantly, negligible at zero magnetic field, confirming the antiferromagnetic nature of the sample. These results are consistent with previously reported *M*-*H_z_* curves of Co_1/3_TaS_2_.


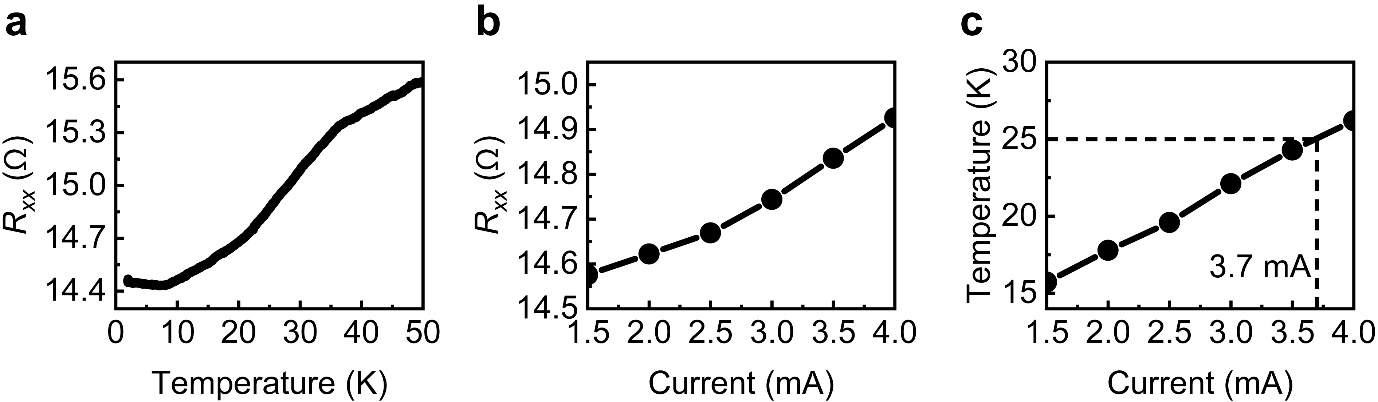


**Figure S4.** Quantification of the Joule heating effect. a) *R_xx_*-*T* curve of the CoTaS_2_ device measured with a reading current of 0.1 mA. b) *R_xx_*-*I* curve of the CoTaS_2_ device measured at 15 K. c) Estimated sample temperature from (a) and (b). A writing current exceeding 3.7 mA raises the sample temperature above *T*_N_ of 25 K. Therefore, all switching currents were kept below 3.7 mA to ensure the device remained in the antiferromagnetic phase during the switching experiments.


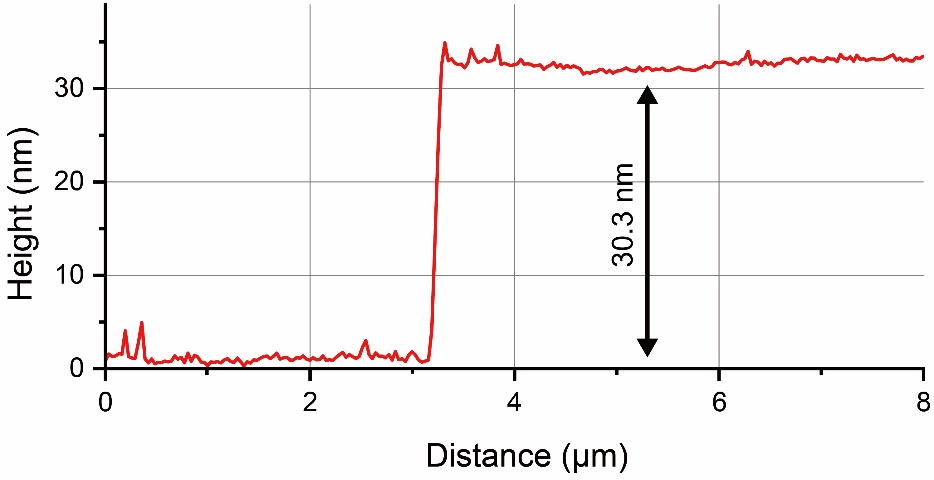


**Figure S5.** Thickness information of the Co_1/3_TaS_2_ device used for the experiments in Fig. 3. The thickness is measured to be about 30.3 nm by the atomic force microscopy.


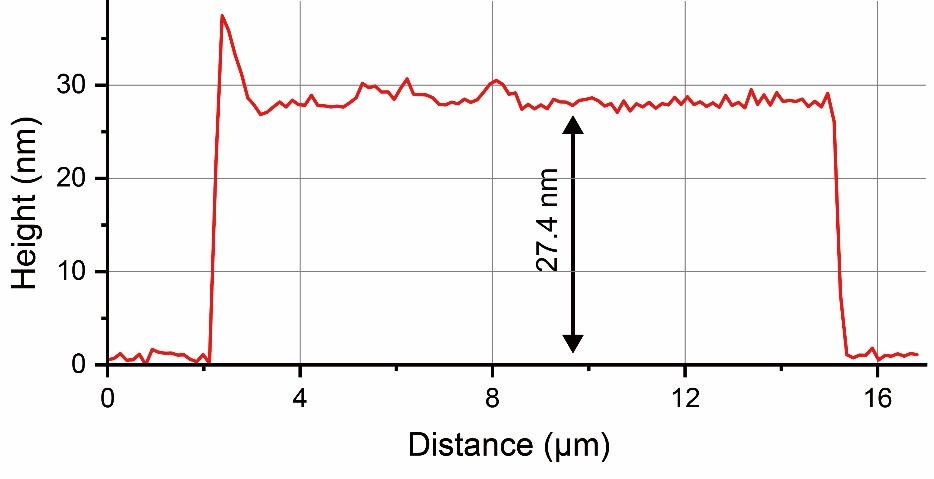


**Figure S6.** Thickness information of the Co_1/3_TaS_2_ device used for the experiments in Fig. 4. The thickness is measured to be about 27.4 nm by the atomic force microscopy.
